# Companies’ Health Technology Assessment Strategies and Practices in Australia, Canada, England, France, Germany, Italy and Spain: An Industry Metrics Study

**DOI:** 10.3389/fphar.2020.594549

**Published:** 2020-12-03

**Authors:** Ting Wang, Neil McAuslane, Lawrence Liberti, Helga Gardarsdottir, Wim Goettsch, Hubert Leufkens

**Affiliations:** ^1^ Centre for Innovation in Regulatory Science (CIRS), London, United Kingdom; ^2^ Division of Pharmacoepidemiology and Clinical Pharmacology, Utrecht Institute for Pharmaceutical Sciences, Utrecht University, Utrecht, Netherlands; ^3^ National Health Care Institute, Diemen, Netherlands

**Keywords:** metrics, reimbursement, health technology assessment, market access, drug development

## Abstract

**Background:** Health technology assessment (HTA) has increased in importance in supporting payer decision making by assessing the relative effectiveness and cost effectiveness of new medicines. Thus, pharmaceutical companies need to address the HTA requirements early during development to improve reimbursement outcomes. Currently, there is a lack of research to assess the impact of HTA on development and jurisdictional outcome from companies’ perspectives. This study aimed to assess companies’ HTA strategy and characterise HTA practice in seven jurisdictions.

**Methods:** A multi-year, annual study collected information for individual products, focusing on development activities regarding inclusion of HTA requirements and selection of global comparators. The generation of local contextual information, submission strategies and predictability of HTA outcomes was examined jurisdictionally in Australia, Canada, England, France, Germany, Italy and Spain. The study questionnaire was built into a secure online data collection platform and data were provided annually by participating companies.

**Results:** Data for 169 compounds were provided by nine international companies between 2014 and 2018. HTA requirements were implemented in evidence generation plan for 63% of products during development. Global comparators were accepted by HTA bodies for more than half of studied products; Spain showed the highest acceptance rate (85%). Companies took advantages of parallel process in Australia and Canada to shorten product rollout time. Australia demonstrated general consistency in HTA review time, and England had the longest variation (interquartile range, 216 days). Requirements for additional information after submission occurred at all HTA bodies. Germany and Italy showed the highest percentage of products being reimbursed as per regulatory label (80 and 68%, respectively). Canada was the most predictable jurisdiction, with the highest proportion of review outcome (90%) that met companies’ expectations.

**Conclusion:** Companies are addressing HTA requirements during development for many products; however, they are challenged by varying requirements and practices and product success ultimately depends on how HTA organisations and payers assess added value in the context of the national healthcare systems. This ongoing study created a baseline to help capture fact-based changes for company HTA strategies and HTA body practices.

## Introduction

Drug development is a long, costly and complex process (DiMasi et al., 2016) and in response to competitive pressure, pharmaceutical companies continue to improve research and development productivity to bring innovative medicines to market (Cohen, 2005; Smietana et al., 2015). There is also a growing interest from regulatory agencies and heath technology assessment (HTA) bodies to adapt flexible processes to expedite the availability of medicines to address critical healthcare needs (McAuslane et al., 2019). Over the last decade, the number of medicines that have received regulatory authorisation has risen, and with 60 approvals in 2018, the US Food and Drug administration (FDA) had its highest number of approvals in the decade (Rodier et al., 2019). However, the success of these products for pharmaceutical companies remain to depend on how HTA organisations and payers will assess their added value in the overall context of the national healthcare systems (Sood and de Vries, 2009).

HTA has increased in importance in supporting payer decision making by assessing the relative and cost-effectiveness of new medicines in comparison to existing technologies based on local context (Goodman and Ahn, 1999). One study showed that only a proportion of regulatory approvals received an initial positive HTA recommendation (Wang et al., 2019), which could result in price constraints, reimbursement restrictions by the payer and time delay to patient access, particularly as new products might become available in different jurisdictions at different times. Therefore, pharmaceutical companies need to address the expected HTA requirements during drug development in order to improve the HTA outcome and to maximise patient access and commercial success.

To this end, companies have implemented cross-functional collaborations within their organisations to bring clinical, regulatory, health economics and outcomes research (HEOR) and access teams together during the drug development process to ensure the generation of evidence that supports both regulatory approval and an HTA recommendation (van Nooten et al., 2012; Wang et al., 2018). Nevertheless, results of a recent stakeholder survey showed that companies were concerned about uncertainties regarding how best to incorporate HTA requirements early in development. Complexities included the variability in HTA requirements across jurisdictions, rapid changes in clinical practice and standard of care that could impact the choice of comparator and often highly divergent economic environments (Wang et al., 2018). Researches have been undertaken to compare the processes and methodologies use by HTA bodies and their recommendations (Schwarzer and Siebert, 2009; Kristensen and Gerdhaus, 2010; Kleijnen et al., 2012; Nicod and Kanavos, 2012; Allen et al., 2014; Lipska et al., 2015; Salas-Vega et al., 2016; Akehurst et al., 2017; Allen et al., 2017; Nicod, 2017; Angelis et al., 2018; Vreman et al., 2020). Table 1 summarises the feature of key HTA agencies studied by researchers. These studies have contributed to the awareness and identification of divergences in HTA recommendations and have reinforced the argument of the need to bring alignment across HTA bodies as an approach to improving patient access to new medicines on a global scale.

**TABLE 1:** Summary of key features of HTA agencies.

Jurisdiction	Regulatory approval	HTA assessment and appraisal	Main HTA criteria	Influence of HTA on drug pricing	Managed entry scheme
Australia	National	National	Clinical, cost effectiveness	Indirectly as it has an impact on ICER	Yes
Canada	National	National and regional	Clinical, cost effectiveness	Indirectly as it has an impact on ICER	Yes
England	Pan- European	National	Clinical, cost effectiveness	Indirectly as it has an impact on ICER	Yes
France		National	Clinical	Yes, ASMR rating used for pricing negotiation	Yes
Germany		National	Clinical	Indirectly through the level of added benefit	No
Italy		National and regional	Clinical, budget impact	Yes	Yes
Spain		National and regional	Clinical, budget impact	Yes	Yes

Notes: Allen et al. (2017) and Angelis et al. (2018).

Works are in progress to promote better alignment of HTA. Early scientific advice programmes have been used as a platform at both national and international levels, for companies to gain insights on the evidence requirements from HTA bodies. A high level of agreement on the evidence generation between EMA and European HTA bodies have been observed during these advice meetings (Tafuri et al., 2016).

In Europe, a proposal for a “Regulation of the European Parliament and of the Council on health technology assessment and amending Directive 2011/24/EU” was published in 2018, suggesting joint work on HTA at Union-level (European Commission, 2018). This proposal was welcomed by pharmaceutical companies as a way to ensure consistency, transparency and synergies in clinical assessment by member state HTA bodies (European Federation of Pharmaceutical Industries and Associations, 2018). The European network for Health Technology Assessment (EUnetHTA) has developed the HTA core model as a standardised framework for the generation of HTA information (EUnetHTA, 2016). This methodological framework has been evaluated by companies and has been found to be useful in improving the efficiency of evidence generation (Gyldmark et al., 2018). In particular, the clinical domain of the core model has been found to be the main driver for HTA recommendations and the consistency that this model brings is expected to support the proposed joint assessment of the clinical value of new products at the European level (Giuliani et al., 2018).

Despite the continued refinement of HTA processes and methodologies, pharmaceutical companies continue to explore the most efficient internal practices that can be implemented during the drug development process to ensure that the best data can be obtained to address jurisdictional HTA expectations, in order to support positive and timely reimbursement outcomes. Currently, there is a lack of research from the companies’ perspective into the impact of HTA requirements on the drug development plan and subsequent jurisdictional submissions and assessments. This study aimed to characterise the practices of international pharmaceutical companies that address HTA requirements by collecting specific metrics and activities for new products from development to rollout at the jurisdictional level. The objectives of this study were to:

- Identify companies’ HTA practices during development and before jurisdictional submission;
- Capture rollout milestones that help provide an understanding of the companies’ submission strategy and HTA bodies’ consistency;
- Examine the predictability of reimbursement outcome.

## Methods

### Development of the Study Questionnaire

A multi-year, annual metrics study was developed by the Center for Innovation in Regulatory Science (CIRS) in partnership with pharmaceutical companies. The development of a study questionnaire evolved in three phases: First, an industry task force of interested senior executives from 7 multinational pharmaceutical companies guided the creation of the initial study proposal. A call for interest was then distributed to 15 multinational companies and 10 companies agreed to participate in the pilot study and took part in a questionnaire development process through a one-day industry discussion meeting. The meeting was held in March 2011 to agree on the methodology and to define the scope of the study, including the jurisdictions and products to be evaluated. The pilot study was conducted during July–September 2011 to collect information on three new active substances (NASs) from each company recently licenced in targeted jurisdictions. This phase identified the metrics to be collected to understand the impact of HTA requirements on the development programme, to assess the rollout timeline of products across jurisdictions and to provide participants with early insights.

Results of this study enabled the refinement of the methodology for next pilot study. The scope of 2012 pilot was expanded to include both recently licenced products and projects currently under pivotal trial development. The inclusion of development projects captured current HTA strategies for drug development and enabled continuous data collection in future studies when the projects become licensed. These pilot studies led to the finalisation of the annual study questionnaire, which has been in use from 2013 onward.

### Structure of the Study Questionnaire

The final study questionnaire was organised into two sections and collected metrics on drug development and jurisdictional roll out. The structure and the rationale of the questionnaire are listed in Table 2.

**TABLE 2:** Structure of the questionnaire.

Sections		Key topics	Rationale		Example questions
Drug development section: 10 questions (28 metrics). Current data collection for development projects; retrospective data collection for licensed products		Compound characteristic and development milestones	Basic characteristics of compound were collected to facilitate tracking the success of the project over the long term and identify different HTA review trends by product type		Active substance type: please select if the product is new active substance or major line extension
					Therapeutic area: Provide the first two levels of the WHO Anatomical therapeutic Chemical (ATC) classification system (enables partitioning of the data by indication)
			Key milestones from development phase were collected in order to determine the relative length of development and whether inclusion of HTA considerations or timing of HTA advice influences development time or decision making		Pivotal trial date: Provide the development milestone of first pivotal dose for the respective indication
		HTA scientific advice	The collection of data on scientific advice meetings during drug development and the impact of these meetings on trial design are important elements in development decision making		When and from whom were companies seeking scientific advice?
					Did the advice change the evidence programme?
		HTA-related considerations	A key element of the development survey is to determine which HTA technical requirements are currently being included in pivotal trials and the extent of their implementation or non-implementation		Which HTA technical requirements have been incorporated into global development? For example, HTA accepted primary endpoint
					Were active comparators/interventions included in drug development?
Jurisdictional section: 9 questions (34 metrics): Jurisdictions included: Australia, Canada, England, France, Germany, Italy, and Spain. *Data collection for recently licensed products*	Local submission strategy			To identify if additional clinical evidence specific to the requirements of the individual jurisdiction were generated and whether any of the local jurisdictions were consulted by the company prior to submission	Was additional local contextual information (in terms of local population and local standards of care) generated prior to submission?	
					When and from whom were companies seeking pre-submission advice?	
	HTA review characteristics			To review the evidentiary package submitted to the jurisdiction, as well as explore specifically the issue of inclusion of comparators into the evidentiary package	Were the comparators in the global evidence package accepted by the local HTA agency?	
				To assess the expectation in terms of reimbursement outcome for each jurisdiction and used to identify jurisdictions that are or are not predictable	Were additional comparator(s) required?	
					What type of additional comparator(s) were required?	
	Review milestones, HTA appraisal/reimbursement outcome			The dates for product submission to respective agencies and the dates of agency decisions will be used to compare the timeliness and the consistency of different agencies	First HTA submission and recommendation date	
					Final coverage decision date	
				Compare the recommendations of the key HTA/decision-making agency in each jurisdiction, as well as the final reimbursement outcome	Reimbursement label to the regulatory approval label population	
					Post-marketing studies requirements	
	Reason for success and outstanding issues			To ascertain reasons for success for each product and to identify issues of concern that were raised, irrespective of recommendation outcome, by the HTA agency	Provide information on the outstanding technical issues raised by agency during the HTA decision-making process	

### Product and Jurisdiction Inclusion Criteria

The scope of products in the study covers both projects under development and licenced products. Information for both NASs and major line extensions (MLEs) that met the criteria were collected. The inclusion criterion for the development projects were pivotal trials beginning within 1 year from the data collection year. The inclusion criterion for the licenced products were market authorisation or HTA recommendation in a target jurisdiction within 1 year from the data collection year. There is no restriction on the therapeutic area, all compounds fit the above criteria have been included in the study.

Exclusion criteria were: generics; vaccines; development of a marketed active substance without any change to formulation or indication/disease state; changes to labelling for reasons other than those relating to new indications/disease states or new formulations; changes to manufacturing and control methods; applications where a completely new dossier was submitted from a new company for the same active substance and the same indication(s) as already approved for another company; and applications from a new or additional name, or a change of name for an existing compound.

The key jurisdictions included in the study were Australia, Canada, England, France, Germany, Italy, and Spain. Jurisdictions were selected by study participants based on the importance of the market to companies and the maturity of the HTA systems. For Canada, Italy and Spain, data on HTA were collected at the national level, the regional adoption of national HTA decisions was out of scope of this study.

### Milestone Definitions

“First worldwide regulatory submission” was defined as the date a product was submitted to the first regulatory agency for market authorisation anywhere in the world. “Regulatory submission gap” was calculated as the time taken from first worldwide regulatory submission to the submission to local regulatory agency. “Regulatory review time” was defined as the time taken from the submission of the dossier to the approval by the specific regulatory agency [EMA review time was defined as the date of application submission to the date of the European Union (EU) Commission decision]. “HTA submission gap” was defined as the time taken from the date of local regulatory approval to the date of the first submission to the jurisdictional HTA body. “HTA review time” was defined as the time taken from the first submission of the value dossier to the date of the first HTA recommendation in that jurisdiction, HTA review time for re-submissions was not included in this analysis.

### Data Processing and Analysis

The study questionnaire was built into a secure online data collection platform developed by CIRS, and data were provided by company participants during second and third quarter each year. Data collection was completed by the third quarter each year and the data were exported into an Excel file and analysed using descriptive statistics. For each analysis reported in this paper, the cohort of products included in the calculation was based on the completeness of data provision. To maintain confidentiality, only aggregated results were reported and any data that identified an individual product or a specific company were excluded from the analysis.

In the timeline analysis, median time in days was calculated for products rolled out to each jurisdiction; the range of HTA review time was also explored using a box plot to show the variation between 25th and 75th percentiles; product characteristics such as NAS type and main therapeutic area were applied to stratify analysis results.

Jurisdictional predictability was studied based on variation of HTA review time and level of expectation in HTA recommendation. The HTA review time measured the time taken from submission to first HTA recommendation, regardless the outcome of the recommendation. The review time variation of each jurisdiction was analyzed by the interquartile range of HTA review time for all products assessed in the jurisdiction. The expectation of HTA recommendation was subjective measure of companies’ view, companies were asked to rate if the recommendation was expected or not, regardless of the outcome of the recommendation. The level of expectation in HTA recommendation was calculated based on the number of products for each jurisdiction that achieved the company’s expectation among all products assessed in that jurisdiction.

## Results

In this paper, we excluded data from the pilots and focused on information provided by companies that participated between 2014 and 2018. A total of 169 compounds were collected from nine international companies during this period, of which 66% were NASs. More than half of the compounds (53%) in the database were oncology products, which were consistent with the top therapeutic areas identified in the current development pipeline and recently approved products (Albrecht, 2018). The jurisdictional information was analysed based on licenced products and the timing of first worldwide regulatory submission for those products ranged from November 2006 to August 2017. For each analysis in this paper, the number of products assessed at jurisdictions varied due to the availability of data for that question, the number of products and companies were stated in each figure.

### Evidence Requirements During Drug Development and Rollout

For 65 of 104 licenced products (63%), HTA requirements were considered and implemented in the evidence generation plan, which showed a good level of incorporation of HTA expectations during development. However, practices varied between companies, ranging from 37% to 100% of the developed products, showing different strategies among the participating companies.

The most commonly included technical HTA requirements among the 65 products were safety measures (92%), HTA acceptable secondary endpoints (89%), patient selection criteria (88%), study design elements (88%), HTA acceptable primary endpoints (86%) and trial duration (85%). Non-technical requirements were also embedded, including addressing the place of the new therapy in treatment pathways (75%), addressing unmet medical need (71%), and providing a cost-effectiveness evaluation (65%). We followed up the comparators included in the global development plan by companies and investigated the acceptance of the comparator choice by HTA bodies during roll out.

**FIGURE 1:**
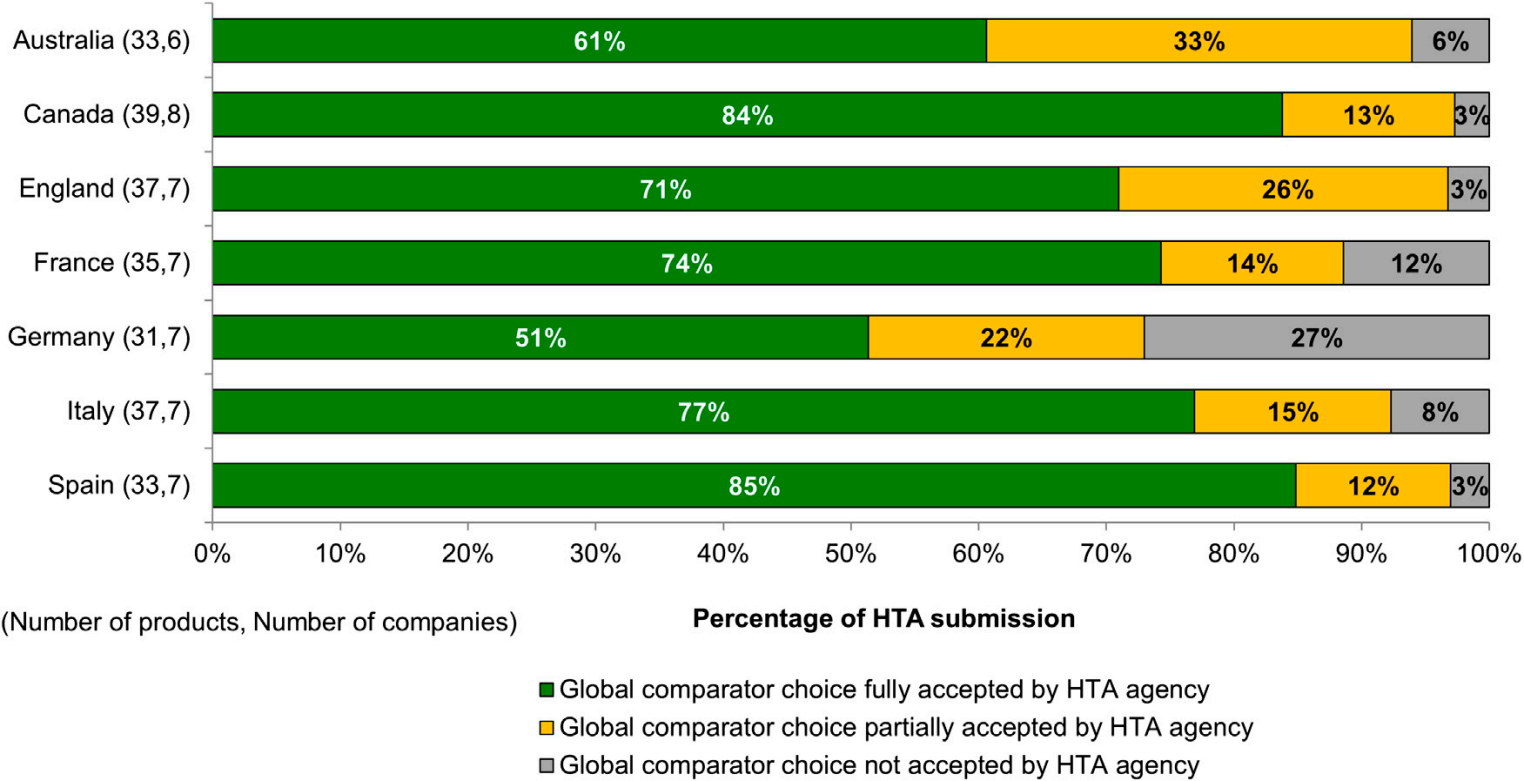
Acceptability of companies’ selectison of comparators in global clinical trials.

For more than half of the submissions, the choice of the comparator was fully accepted at target HTA bodies, with Spain and Canada showing the highest acceptance rate (Figure 1). In some cases, HTA bodies also partially accepted the global comparator choices, and requested additional comparators to their assessment. This was seen mostly in Australia (33% of submissions) and England (26% of submissions). HTA bodies that conducted benefit assessment (e.g., in France and Germany) showed the highest proportion of comparator rejections, 12% and 27% of total submissions, respectively. For submissions where the global comparators were not accepted, additional comparators were required by the HTA bodies. In most cases (77%) comparators based on the local standard of care for this indication were requested, and 23% of cases recommended the use of the least costly therapy as the comparator.

In this study, eight products were reviewed in all seven target jurisdictions, however, their reimbursement status varied across all jurisdictions. Four of the eight products had their global comparators accepted (full or partially) across all seven jurisdictions, nevertheless in the case of the other four products, the comparator choices were not accepted by one or two HTA bodies.

In addition to the evidentiary package based on the global development plan, we observed that companies in this study generated local contextualised information before submission to meet the specific requirement of an HTA body. A high proportion of submissions to England (90%) incorporated local contextual information (in terms of local population and local standard of care), followed by Germany (82%), Italy (80%), Spain (79%), France (72%), Canada (63%) and Australia (61%).

**FIGURE 2:**
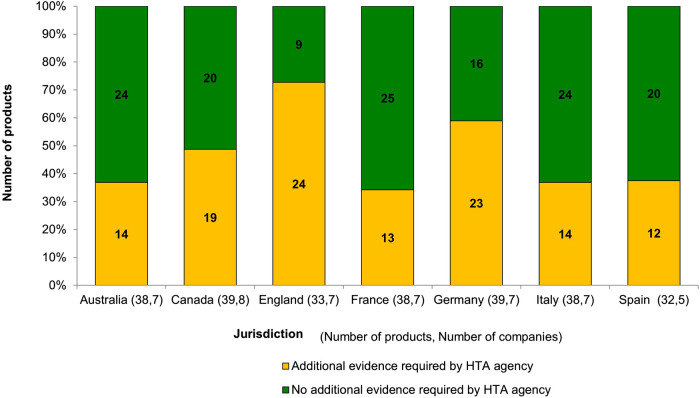
Proportion of companies’ submissions where additional evidence were requested.

The study revealed that after the dossiers were submitted, HTA bodies still required additional evidence to be provided by the companies to support the assessment. Figure 2 showed the proportion of submissions at the local level for which additional evidence was required by HTA bodies. England showed the highest frequency of requesting additional evidence from companies, with 63% requests being for a locally relevant comparator; this was followed by Germany, with 56% requested being sub-group analysis. We further analysed the details of the evidentiary requests across all HTA bodies: 53 of 120 requests (44%) were related to the use of a locally relevant comparator, 35% were for a sub-group analysis, 26% were for a locally relevant economic analysis, 24% were to contextualise the evidence to the local population, 21% were for the use of a different analysis methodology, 13% were related to the use of a network meta-analysis, and 10% were requests for trial data in the local population.

### Companies’ Submission Strategy to Regulatory Agencies and Health Technology Assessment Bodies

Products that received HTA recommendation in targeted jurisdictions were analysed for their rollout time, that is, the time taken from first regulatory submission to the HTA decision in each local jurisdiction. Companies were likely to submit to Europe for market authorisation first across the target jurisdictions, followed by Australia and Canada, with median delays of 81 and 73 days, respectively.

In Australia and Canada, companies can submit the dossier to the respective HTA body before the market authorisation is granted; the median overlap between the regulatory and HTA process was 107 days in Australia and 30 days in Canada. There was a variation from the EMA approval to the HTA submissions in Europe; the median time gap was 7 days in England, 23 days in Italy, 29 days in France, 42 days in Germany and 49 days in Spain. Companies sought advice from agencies before HTA submission, the study showed that Germany has the highest proportion of pre-submission advice among its total submissions (73%), followed by Australia (69%), France (35%) and Canada (23%). Information on pre-advice in other jurisdictions was limited.

**FIGURE 3:**
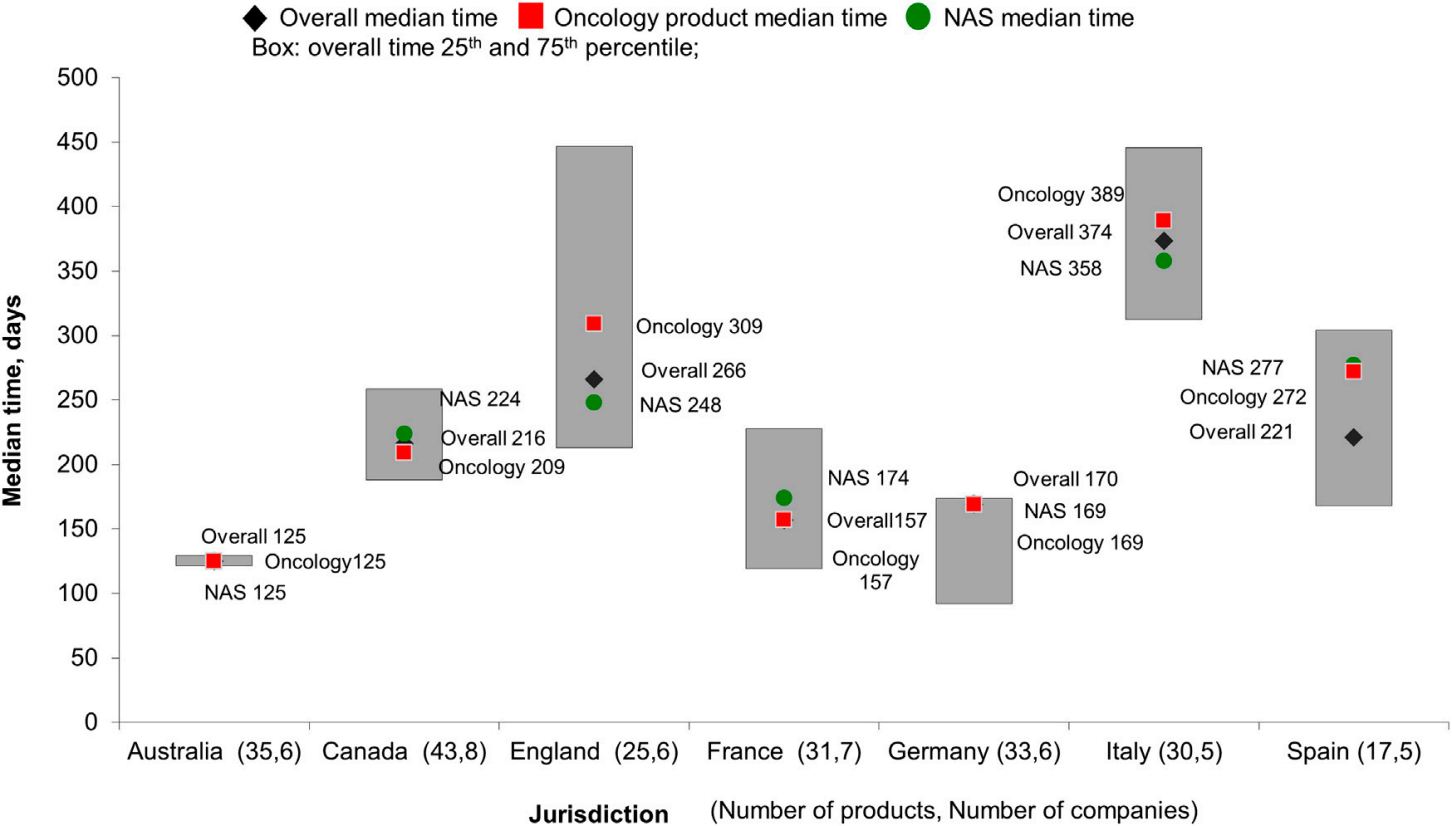
HTA review time for products provided by participating companies.

The time from HTA submission to recommendation varied across the targeted European jurisdictions, ranging from 155 days in France to 375 days in Italy. Figure 3 illustrates the median time and 25th to 75th percentile of HTA review for products provided by companies in each jurisdiction. Australia demonstrated general consistency in HTA review time, with interquartile range (IR) being 9 days. England had the longest variation for HTA reviews (IR, 216 days), followed by Spain and Italy (IR, 161 and 144 days respectively). Canada and Germany showed similar variation in the review process with IR being 97 and 89 days.

We further stratified the HTA median review time by product types. For companies that submitted oncology products for HTA review, the median time taken to receive HTA decision was longer in Spain, England and Italy compared with overall median time; there were no differences in median time to receive HTA decision for oncology products in Australia, France and Germany. The biggest divergence in HTA review time for oncology products was observed in Spain, where it was 51 days longer than the overall median. Interestingly, Spain also showed the biggest difference in median HTA review time for NASs compared with overall products, which was 56 days longer. In England and Italy, NASs products were reviewed faster (40 and 6 days, respectively) compared with the overall median.

### Companies’ Predictability of Health Technology Assessment Success and Restriction on Reimbursement

Predictability of HTA outcome plays an important role in market access planning for companies. In this study, participating companies were asked if the outcome of the HTA recommendation for each of their products had achieved the companies’ expectation prior to submission. France was identified as the least predictable jurisdiction, based on the outcome of the initial HTA recommendation (55% of total submissions), followed by Italy (58%) and Germany (70%). In comparison, Canada showed the highest proportion of products (90%) that met companies’ initial expectation regarding HTA outcome.

**FIGURE 4:**
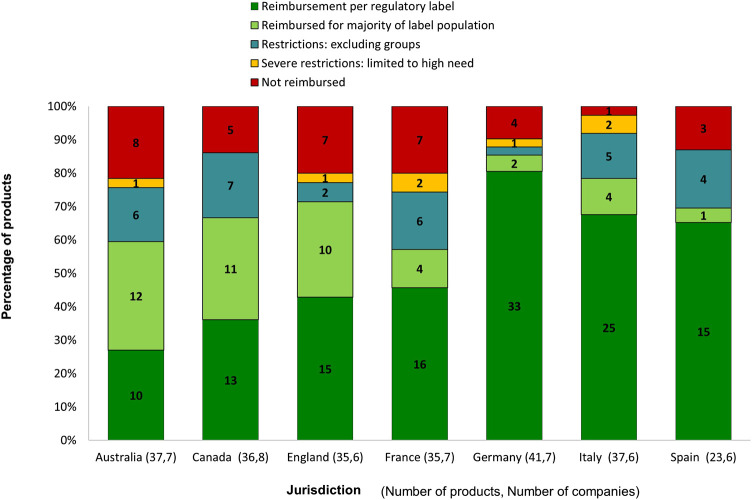
Reimbursement decisions for products provided by participating companies.

In relation to the reimbursement outcome, we assessed the reimbursed indication by comparing it with the authorised label use (Figure 4). Germany and Italy showed the largest proportion of products reimbursed as per regulatory label, while Australia applied the highest percentage of label limitations (72%) to its submissions. In Germany, four products were reviewed as “no added benefit” and were subsequently withdrawn by the companies. The four products were categorised as “not reimbursed.” No product in this study received the same initial reimbursement outcome across all jurisdictions.

For products for which the companies indicated that they had an expected HTA outcome, the majority (93%) were reimbursed fully or with restriction to label population. Meanwhile, for products that were not reimbursed or severely restricted of use, 70% of their HTA outcomes were viewed as “unexpected” by companies. In this study, 55 reimbursement decisions were granted with staged entry to market, which was mostly used in Australia (38% of reimbursement decisions), Italy (32% of reimbursement decisions) and Canada (25% of reimbursement decisions). The most utilised mechanisms were “risk-sharing plan required for reimbursement” (47%) and “managed entry scheme” (35%).

## Discussion

A clear understanding of how HTA requirements are embedded in drug development and addressed in jurisdictional submissions is imperative for companies to ensure better predictability of an HTA outcome. This study collected HTA related metrics for individual products from companies, the results provided a snapshot of companies’ current practices in terms of including HTA requirements in evidence generation plan, submission strategy to HTA bodies and their predictability of HTA success. The results also reflected the divergences of HTA systems from companies’ perspective and provided practical implications for companies to improve the understanding and readiness for jurisdictional HTA submission.

### Companies’ Practice in Generating Health Technology Assessment-Relevant Evidence During Development and Rollout

First, this study evaluated the acceptance of comparator choice by HTA bodies. Clinical trials provide an important evidence base for regulatory and HTA assessments. It is important for companies to choose the right active comparator in the development phase to ensure the scientific validity of trial designs and to be able to prove the value proposition of new products. Our results revealed a good level of acceptance on comparator amongst the HTA bodies studied, reflecting that companies were generally making the right development decisions. A survey conducted in 2017 among HTA bodies in Europe confirmed that the efficacy and safety profile were the most important criteria for comparator choice, along with identifying the comparator that was likely to be replaced by the assessed technology (Kristensen, 2017). However, companies in our study were challenged in Germany with a 27% rejection rate on the global active comparator choice. This may be because the added benefit of new medicines was assessed on subsets of the population by Institut für Qualitaet und Wirtschaftlichkeit im Gesundheitswesen (IQWIG) (Kaiser et al., 2015); therefore, additional comparators were utilised to identify benefits in the subgroups. A better understanding of the rationale for comparator selection by different HTA bodies is, therefore, needed. The choice of comparators has been a key discussion component at EMA-HTA parallel advice meetings; divergences were observed in the advice provided across different HTA bodies, and the potential solution of using indirect comparison was recognised (Tafuri et al., 2016).

Second, this study evaluated the companies’ preparation before the HTA submission at the jurisdictional level. Local evidence generation related to comparisons to the local population and local standard of care was seen often in submissions to England and Germany. This suggested that the local company affiliates in these jurisdictions were actively preparing for the HTA submission, translating the global evidence package to the local context. Conversely, the highest proportion of HTA submissions requiring additional evidence were in England and Germany, which showed a divergence between companies’ and HTA bodies’ perspectives. In Germany, the most requested information after the HTA submission was a subgroup analysis. This issue has been recognised by other researchers and a more comprehensive discussion between companies and HTA bodies was suggested regarding the meaningfulness of subgroup analysis (Rasch and Dintsios, 2015). It has been recognised that a minimum set of evidence requirements could be prepared for HTA submission across Europe (Oyebode et al., 2015); however, to move forward with a centralised HTA assessment in Europe, it is crucial to understand the additional evidence required among HTA bodies, why these requests diverge across the jurisdictions, and the ultimately added value of extra evidence generation.

### Health Technology Assessment Submission Strategies and Rollout Timelines

Timely recommendations for drug reimbursement by HTA bodies is critical to ensure patient access to new medicines. Researchers continuously monitor HTA timelines as an indicator of drug availability (Wang et al., 2019; Zamora et al., 2019); however, because HTA submission dates are not generally publicly available, these studies have been based on milestones collected from the public domain and have only measured the overall time from regulatory approval to the HTA recommendation. As the milestone metrics in this study were provided directly by companies and included the HTA submission dates, our rollout analysis was able to illustrate the full picture of regulatory and HTA pathways in the key jurisdictions.

In Australia, a parallel review process has been available since 2011 for companies to submit HTA dossiers prior to receiving market authorisation. Although the process allows companies to submit HTA dossier to the Pharmaceutical Benefits Advisory Committee (PBAC) as soon as the regulatory application to the Therapeutic Goods Administration (TGA) is accepted for review, but HTA decisions cannot be made until the TGA delegate report is finalised for approval (Pharmaceutical Benefit Scheme, 2018). Our data showed that companies generally submitted a median of 107 days prior to the TGA regulatory approval and consequently, Australia was typically the first country in which companies received an initial HTA recommendation within the studied jurisdictions. The parallel process has also been available in Canada since 2012; it differs from the Australian system in that submission to the Canadian Agency for Drugs and Technology in Health (CADTH) should occur within 90 days before the date of anticipated notification of compliance (NOC) from Health Canada. In our study, companies tended to submit the HTA dossier approximately 1 month prior to the regulatory approval in Canada. From April 2, 2018, the deadline for CADTH submission was extended from 90 to 180 days before the anticipated NOC (Canadian Agency for Drugs and Technologies in Health, 2020). It is expected that the impact of this extension on companies’ submission strategies will be reflected in future results from this continuing study.

The submission gap from EMA approval to submission to European HTA bodies can be attributed to both company submission strategies and HTA system settings. In England, companies are likely to generate local contextual evidence prior to the HTA submission and the submission gap showed in our study was only 1 week (median). This may be because National Institute for Health and Care Excellence (NICE) conducts scoping exercises before a product has received a market authorisation and before an appraisal topic is referred to NICE by the Department of Health (National Institute for Health and Clinical Excellence, 2009).

In Germany, the HTA process starts within 3 months from regulatory approval by law, and the HTA assessment is to be completed within 6 months from submissions (Gemeinsamer Bundesausschuss, 2020). In our study, the submission gap was a median 42 days (1.4 months) in Germany, and HTA review time was a median 170 days (5.7 months), showing good compliance with these defined timelines.

In general, HTA submissions were conducted across all the studied European HTA bodies within 2 months of EMA approval, showing that it is possible for companies to submit the HTA dossiers in a timely manner. This supports the case that companies can be ready to submit their value dossiers quickly should a centralised HTA platform come into play in the near future.

The variation in HTA review timelines can be explained by the different review procedures used and the nature of company interactions during the review. The median HTA review time in Australia was consistently 4 months, which reflected the frequency of the PBAC Committee meeting; the timeline did not differ for NASs and MLEs, or by therapeutic areas, and this consistency confirmed that HTA in Australia was procedurally predictable.

Company-HTA body interactions during assessment such as providing additional evidence and clarifications on questions can contribute to longer HTA review time. A number of HTA bodies applied a stop-the-clock mechanism during the HTA process (Kristensen, 2017), for example, in England, NICE will allow a clock stop for certain products. In our study England showed the most variation in review time, which was also in line with the high proportion of requests for additional evidence. Despite that the observation that Germany requested additional evidence for a high proportion of its submissions, the review time was within 6 months, in compliance with the law. Certain HTA bodies employed a clock-stop mechanism while companies were preparing a response; we did not characterise whether the clock-stop was applied by the studied HTA bodies. Companies also sought pre-submission advice from HTA agencies, such activities are intended to improve the quality of the dossier submitted and potentially reduce the need for clarification during the assessment. Further research is needed to assess the link between pre-submission advice and company-HTA interaction during the assessment.

In England, the HTA review of oncology products took longer than the median NICE review time; in the case that NICE appraisal concluded that there was insufficient evidence to support a recommendation, products could be reimbursed through cancer drug fund (National Institute for Health and Clinical Excellence, 2020).

### Practical Implications for Companies

HTA bodies are continuously improving their procedures and methodologies to ensure quality decision making that enables timely patient access to medicines of value. Research has been carried out to identify attributes that underpin a good HTA submission and review (Mazumder et al., 2015; Wang, 2015). A recent literature review summarised the areas in which good HTA practices have been identified, including the identification and interpretation of evidence, priority setting, framing, scoping principles, and HTA implementation. This research also pointed out areas in which good practices were currently lacking, including defining the organisational aspects of HTA, the use of deliberative processes and measuring the impact of HTA (Kristensen et al., 2019). However, there was no systematic and continuous measure of HTA submission and review practice. Our study collected metrics on individual products from companies and provided unique insights regarding HTA bodies’ review practices by characterising timeliness, transparency and predictability at key jurisdictions.

Australia showed the greatest predictability regarding HTA review time and outcome expectation; the consistent review time of 125 days was associated with the frequency of the PBAC Committee meeting (Pharmaceutical Benefits Advisory Committee, 2017). Moreover, companies have taken advantages of the parallel process in Australia with a median 107-days overlap between regulatory and HTA review, which resulted in shortening the overall rollout time. However, Australia was the country to most often not reimburse medicines as per regulatory label in this study. CADTH, which was the second most consistent HTA body in terms of review time, also showed a high level of acceptance of active comparators used in global clinical trials. Whilst companies need to be aware of additional evidence requirements by CADTH during the review process, which affected half of its submissions in this study; most of the CADTH recommendations met the expectations of companies, reflecting a good understanding and predictability of the system. Medicines were also likely to be reimbursed with limitations compared with the approved regulatory label in Canada.

In England, NICE does not appraise all new medicines approved by EMA; however, the topic selection was transparent, with its rationale, process and decisions published on the NICE website. As part of the topic selection, NICE scoping activity includes a draft scoping report and scoping workshop to identify information related to the medicine before EMA approval. The scoping step was viewed by NICE as a critical step to ensure a successful appraisal (Kaltenthaler et al., 2011) and this efficient process was reflected in our results in terms of the short gap between EMA approval and NICE submission time, as well as a high number of submissions with local contextual information generated before NICE submission. Nevertheless, NICE had the widest variation in review time compared with all studied HTA bodies, reflecting the NICE process which involves stakeholders and public comments on draft guidance before the finalisation of recommendation (National Institute for Health and Clinical Excellence, 2009).

France showed the quickest median HTA review time among all European jurisdictions in this study. However, the speed of decision was compromised by a less predictable outcome, with 45% of applications submitted to Haute Autorité de Santé (HAS) receiving an unexpected benefit rating. A 12% rejection rate of global comparator choice in France also demonstrated the needs for further communication between companies and the HTA body during the development stage to facilitate the local submission and improve the predictability of the outcome.

The German HTA system was consistent in terms of submission gap and review time, and complied with the timeframe of 3 and 6 months respectively as defined in law. The outcome of Federal Joint Committee (Gemeinsamer Bundesausschuss, G-BA) benefit assessment was associated with the price negotiation between companies and the National Association of Statutory Health Insurance Funds (Spitzenverband Bund der Krankenkassen, GKV-SV); therefore, the reimbursed labels of products in our study were mostly in line with regulatory approval. To achieve better G-BA outcome for a favourable reimbursement price, companies need to have a better understanding of the evidentiary requirements in Germany, in particular, regarding active comparator choice and sub-group analysis.

Italy stood out among all studied jurisdictions with the longest HTA review time. Despite the fact that companies submitted dossiers for HTA review just 23 days after EMA approval, it took more than 1 year for products to gain an HTA recommendation in Italy. The duration of the review time may be attributed to the process of price negotiation and access restrictions. AIFA implemented extensive use of outcomes-based managed entry agreements (Angelis et al., 2018), and a 2019 study by Villa et al. showed that the managed entry agreement and product monitoring registry were the main determinants for price negotiation, that led to reduction from the proposed price by industry to the final negotiated price (Villa et al., 2019). In our study, although results showed that 80% of evidence packages submitted for HTA review in Italy included local contextual information and 77% used the comparator choice accepted by HTA, HTA outcomes were still unexpected for 42% of total Italian HTA reviews in this study, and more than one third of HTA recommendations required staged entry to market.

Spain had the highest acceptance rate of comparator choices (97%) and also good predictability of HTA outcome (77% of total submissions). Companies were prepared for the HTA submission in Spain, with 79% of dossiers including local contextual information, however, this preparation may have led to a submission gap after EMA approval, which was the longest in Spain among all studied European jurisdictions.

### Strength of the Study

Although there is an increasing number of studies to compare the HTA process and subsequent outcomes for new medicines, specific metrics to inform company decision making around HTA requirements are limited. This annual metrics study has been developed by CIRS in partnership with multinational companies. This collaborative approach represents the first effort among industry to collect HTA-related metrics by following individual products from development through to an initial reimbursement decision. The results provide unique insights into both companies’ practices regarding HTA during development and reflected the timeliness, predictability and requirements of HTA systems in studied jurisdictions.

### Limitation of the Study

This study collected information from nine participating multinational companies, therefore the results were viewed through the lens offered by these companies rather than the whole industry. However, we believe these companies were representative of international companies and their practices were a good indicator of other companies’ HTA approaches. Caution needs to be taken when interpreting the jurisdictional results, as these were not a reflection of the overall performance of the studied HTA bodies.

For each product, not all metrics in the questionnaire were provided, due to practical limitations of access. Therefore, the completeness of datasets for each question differed, and resulted in small divergences in the size of datasets used for specific analyses in the study. Another limitation is the type of products provided by company, where oncology products made up to 53% of the database in this study. As regulatory and HTA agencies have been increasing the transparency of their decision making, information such as regulatory public assessment reports and HTA recommendation reports have been made available on the public domain. Aligning the information from the public domain and the company-provided data will enhance the completeness of the database and enable further research questions to be addressed.

## Conclusions

This CIRS-industry study is the first consolidated effort to collect metrics to assess the companies’ practice to address HTA requirement during development and rollout. The results demonstrated that companies have been actively including HTA requirements during development and generated local contextual information for jurisdictional HTA review. Companies utilised parallel regulatory/HTA review processes in Australia and Canada, while timing of HTA submission after EMA approval varies in European jurisdictions. The collection of jurisdictional evidence requirements, predictability of HTA outcome and reimbursement decisions provided insights into different approaches of HTA bodies. This ongoing study will create a baseline to help address fact-based changes for both companies’ HTA strategies and the practices of the studied HTA bodies. As the HTA landscape is evolving, these study results will support future convergence of evidentiary requirements across HTA bodies and more aligned process between regulatory and HTA agencies to expedite patient access.

## Data Availability Statement

The raw data supporting the conclusions of this article will be made available by the authors, without undue reservation.

## Ethics Statement

Ethical review and approval was not required for the study on human participants in accordance with the local legislation and institutional requirements. The patients/participants provided their written informed consent to participate in this study.

## Author Contributions

TW, NM, and LL: designed the study, analysed data, wrote the manuscript. HG, WG, and HL: provided guidance for the data analysis, and critically reviewed the manuscript.

## Conflict of Interest

The authors declare that the research was conducted in the absence of any commercial or financial relationships that could be construed as a potential conflict of interest.

## Abbreviations

CADTH, Canadian Agency for Drugs and Technology in Health; CIRS, Center for Innovation in Regulatory Science; EU, European Union; EMA, European Medicines Agency; FDA, Food and Drug Administration; G-BA. Gemeinsamer Bundesausschuss; GKV-SV, Spitzenverband Bund der Krankenkassen; HAS, Haute Autorité de Santé; HEOR, health economics and outcomes research; HTA, health technology assessment; IQ, interquartile range; IQWiG, Institutfür Qualität und Wirtschaftlichkeit im Gesundheitswesen; MLEs, major line extensions; NASs, new active substances; NICE, National Institute for Health and Care Excellence; NOC, notice of compliance; PBAC, Pharmaceutical Benefits Advisory Committee; TGA, Therapeutic Goods Administration.
